# IncA/C plasmid encoding *bla*_CTX-M-55_ in non-O1 *Vibrio cholerae* isolates from the edible river fish *Mastacembelus* sp.

**DOI:** 10.1128/mra.01226-23

**Published:** 2024-02-15

**Authors:** Takahiro Yamaguchi, Michio Jinnai, Doan Tran Nguyen Minh, Oanh Nguyen Hoang, Hien Le Thi, Phong Ngo Thanh, Phuong Hoang Hoai, Phuc Nguyen Do, Chinh Dang Van, Daisuke Motooka, Shota Nakamura, Yuko Kumeda, Atsushi Hase, Tatsuya Nakayama

**Affiliations:** 1 Division of Microbiology, Osaka Institute of Public Health, Osaka, Japan; 2 Department of Microbiology, Kanagawa Prefectural Institute of Public Health, Chigasaki, Kanagawa, Japan; 3 Institute of Public Health in Ho Chi Minh City, Ho Chi Minh City, Vietnam; 4 Genome Information Research Center, Research Institute for Microbial Diseases, Osaka University, Suita, Osaka, Japan; 5 Research Center of Microorganism Control, Osaka Metropolitan University, Sakai, Osaka, Japan; 6 Faculty of Contemporary Human Life Science, Tezukayama University, Nara, Japan; 7 Graduate School of Integrated Sciences for Life, Hiroshima University, Higashi-Hiroshima, Hiroshima, Japan; DOE Joint Genome Institute, Berkeley, California, USA

**Keywords:** ESBL-producing non-O1 *V. cholerae*, IncA/C, *bla*_CTX-M-55_, *Mastacembelus* sp., Vietnam

## Abstract

Extended-spectrum β-lactamase-producing non-O1 *Vibrio cholerae* was isolated from edible *Mastacembelus* sp. in Vietnam. The genome sequence was sequenced using DNBSEQ-G400 and MinION Mk1b. A plasmid of approximately 183-kb encoding *bla*_CTX-M-55_ and *bla*_TEM-1_ was detected.

## ANNOUNCEMENT

The spread of antimicrobial resistance (AMR) is a global threat that needs to be discussed from the perspective of the “One Health” concept. As AMR ha reportedly been found in food microbiomes, the spread of plasmid-mediated AMR through food products should be of concern (1). Sequencing the genomes of antibiotic-resistant bacteria isolated is expected to contribute to infer the origin and to prevent the spread of antibiotic-resistant bacteria. To date, few reports have been made on the complete genome of V*ibrio cholerae* encoding the extended-spectrum β-lactamase (ESBL)-related gene. Here, we report the whole genome sequence (WGS) of ESBL-producing non-O1 *Vibrio cholerae* isolated from purchased edible fish from retail stores in Ho Chi Minh City, Vietnam, in March 2020.

Five grams of fresh *Mastacembelus* sp. fish gut contents were mixed with 45 mL of buffered peptone water (Merck, Darmstadt, Germany). After incubation at 37°C for 24 h, 100 µL of bacterial broth was spread on thiosulfate citrate bile saccharose agar (Shimadzu Diagnostics, Tokyo, Japan) containing cefotaxime (2 µg/mL) and incubated at 37°C for 24 h. Several typical colonies were selected, and *Vibrio* spp. isolates were investigated for antibiotic susceptibility using previously published approaches (2). As a result of analysis, VN152 was identified as non-O1 *V. cholerae*. After subculturing in trypticase soy broth medium at 37°C for 18 h, bacterial DNA was extracted using NucleoBond HMW DNA (Takara Bio, Shiga, Japan) for long- read sequencing and smart DNA prep (Analytik Jena, Kanagawa, Japan) for short-read sequencing according to the manufacturer’s instructions, respectively. The extracted DNA was checked using a Qubit dsDNA HS Assay Kit (Thermo Fisher Scientific, Waltham, USA). The library for short- and long-read sequencing was prepared with MGIEasy FS DNA Library Prep Kit V2.0 (MGI Tech, Shenzhen, China) and Rapid Barcoding Sequencing (SQK-RBK004) kit according to the manufacturer’s protocol, respectively. Short-read sequencing and long-read sequencing were performed using DNBSEQ-G400 (MGI Tech, Shenzhen, China) with a 2 × 200 bp paired-end protocol and Oxford Nanopore MinION Mk1b (Oxford Nanopore Technologies, Oxford, UK) with flow cell R9.4.1 according to the manufacturer’s instructions, respectively. After obtaining short-read sequences (total reads 11,708,392; mean length after filtering 2 × 199 bp reads; total bases 2.33 Gb; coverage 568×), trimming and quality checks were conducted using fastp v0.20.0 (3). Short- and long-read quality checks were performed with fastqc v0.11.9 (4) and NanoPlot v1.38.0 (5), respectively. The long-read sequencing data (total reads 41,730; read length N_50_ 17,270 bp; total bases 421 Mb; coverage 105×) were trimmed and then assembled using flye v2.9.2 (6). Short-read sequencing data were used for polishing the long-read assembly with polypolish v.0.5.0. Annotation was performed using DFAST. The assembled WGS was analyzed for non-O1 *V. cholerae* using TYGS (https://tygs.dsmz.de), MLST2.0, and ResFinder4.1. Default parameters were used except where otherwise noted.

**Fig 1:**
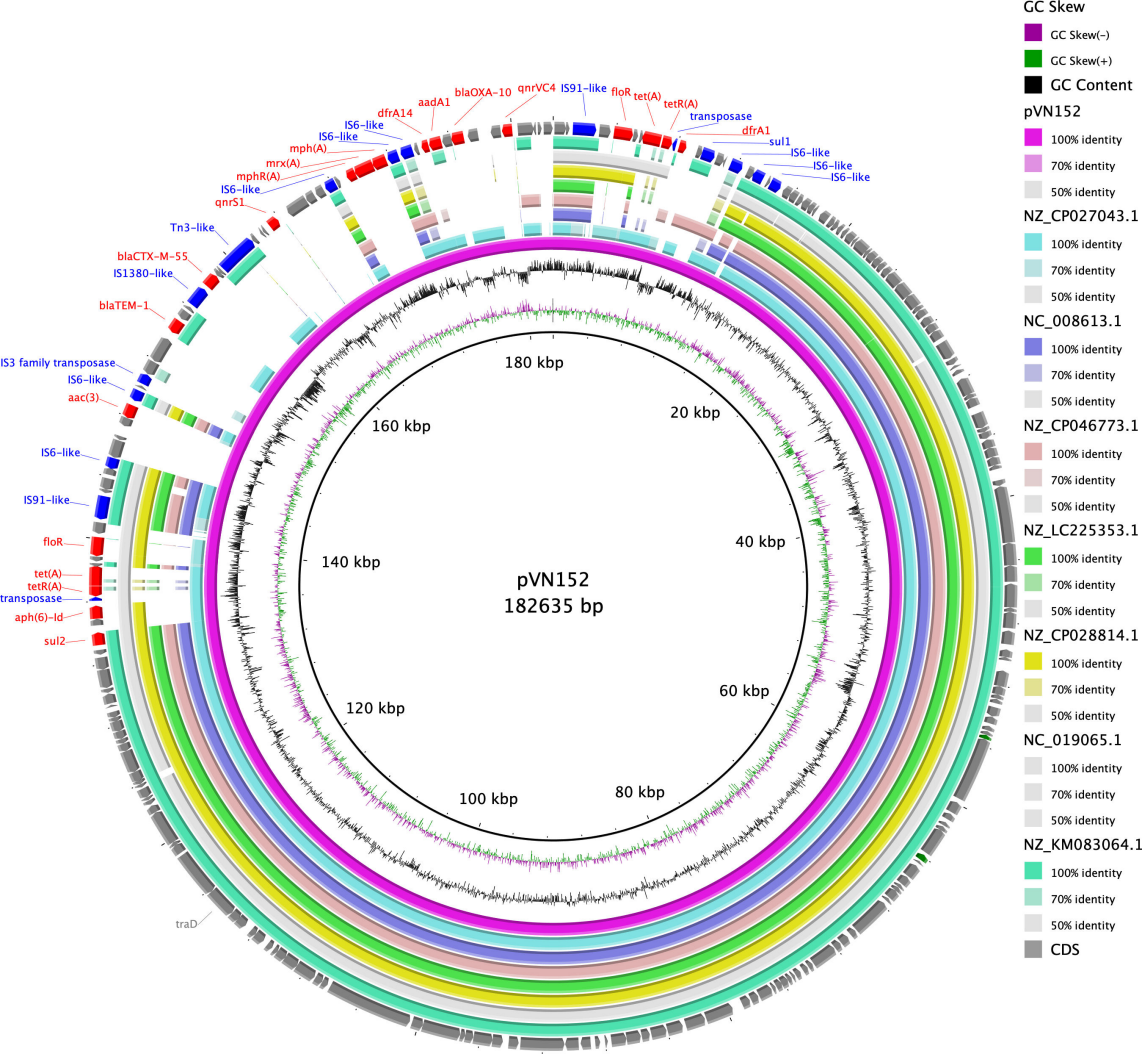
Complete plasmid map of pVN152 from non-O1 *Vibrio cholerae* VN152. Comparison with seven highly similar plasmids (GenBank accession no. NZ_CP027043.1, NC_008613.1, NZ_CP046773.1, NZ_LC225353.1, NZ_CP028814.1, NC_019065.1, and NZ_KM083064.1). Plasmid maps were designed using BLAST Ring Image Generator v0.95.

The assembly resulted in three contigs, carrying two chromosomes (2,873,080 and 1,202,571 bp) and plasmid IncA/C *bla*_CTX-M-55_ (182,635 bp) (Table 1). The assembled hybrid genome was analyzed using TYGS to confirm that *Vibrio cholerae* had been isolated, and this isolate did not have the O1 antigen. The results of ResFinder analysis showed *bla*_CTX-M-55_, *bla*_TEM-1_, *bla*_OXA-10_, *floR*, *tet(A*), *dfrA1*, *14*, *sul1*, *2*, *aph(3″)-Ib*, *aph(6)-Id*, *aac (3)-IId*, *mph(A*), *cmlA1*, *qnrS1*, and *qnrVC4* on plasmid (coverage >95%, identity >95%) (Table 1). The pVN152 was compared with plasmids with high similarity as a result of BLAST (Fig. 1). None of the compared plasmids had *bla*_CTX-M-55_.

**TABLE 1:** Genome information of non-O1 *V. cholerae* VN152

Name	Accession no.	Length (bp)	Gc (%)	Antibiotic resistance gene
Chromosome 1	AP028804	2,873,080	47.9	–^*a*^
Chromosome 2	AP028806	1,202,571	46.7	*qnrVC4*
pVN152 (plasmid)	AP028805	182,635	52.6	*bla*_CTX-M-55_, *bla*_TEM-1_, *bla*_OXA-10_, *floR*, *tet(A*), *dfrA1*, *14*, *sul1*, *2*, *aph(3'')-Ib*, *aph(6)-Id*, *aac (3)-Iid*, *mph(A*), *ant(3″)-Ia*, *cmlA1*, *qnrS1*, *qnrVC4*

^*a*^ –, non-detection.

## Data Availability

The WGS of non-O1 *V. cholerae* VN152 was deposited in DDBJ/GenBank (accession numbers AP028804, AP028805, and AP028806). The raw reads were deposited under the accession numbers DRR513378 and DRR513379 with the BioProject number PRJDB16996.
